# Evaluating the impact of price regulation (Drug Price Control Order 2013) on antibiotic sales in India: a quasi-experimental analysis, 2008–2018

**DOI:** 10.1186/s40545-022-00466-4

**Published:** 2022-10-22

**Authors:** Sakthivel Selvaraj, Habib Hasan Farooqui, Aashna Mehta, Manu Raj Mathur

**Affiliations:** 1 Health Economics, Financing and Policy, Public Health Foundation of India, Gurugram, 122002 India; 2 College of Medicine, QU Health, Qatar University, Doha, Qatar; 3 Indian Institute of Public Health Delhi, Public Health Foundation of India, Gurugram, 122002 India; 4 Institute of Dentistry, Bart’s and The London School of Medicine and Dentistry, New Road, London, E1 2AT UK

**Keywords:** India, Price regulation, Antibiotics, Price control

## Abstract

**Background:**

In India, due to a lack of population-level financial risk protection mechanisms, the expenditure on healthcare is primarily out-of-pocket in nature. Through Drug Price Control Orders (DPCOs), the Indian Government attempts to keep medicine prices under check. The aim of this study was to measure the potential impact of DPCO 2013 on the utilization of antibiotics under price regulation in India using large nationally representative pharmaceutical sales data.

**Methods:**

We used interrupted time series analysis, a quasi-experimental research design to estimate the impact of DPCO 2013 on the utilization of antibiotics in the private sector in India. Indian pharmaceutical sales data set, PharmaTrac from a market research company—All Indian Origin Chemists and Distributors Limited—was used for the study. The data are collected from a panel of around 18,000 stockists across 23 different regions of the country. The primary outcome measure is the percentage change (increase or decrease) in the sales volume of the antibiotics under DPCO 2013, measured in standard units (SUs).

**Results:**

Our estimates suggest that post-intervention (after notification of DPCO 2013) there was an immediate reduction (level change) in the sales of antibiotics under DPCO 2013 by 3.7% (*P* > 0.05), followed by a sustained decline (trend change) of 0.3% (*P* > 0.05) as compared to the pre-intervention trend at the molecule level, but both changes were statistically insignificant. However, in terms of ‘average monthly market share,’ the DPCO 2013 notification resulted in a sharp reduction of 579% (*P* < 0.05) (level change) followed by a sustained increase of 9.5% (*P* > 0.05) (trend change) in the ‘market share of antibiotics under DPCO’ as compared to pre-intervention trend.

**Conclusions:**

The impact of DPCO 2013 in terms of the overall increase in the utilization of antibiotics under price regulation was limited but there was a switch from non-price controlled antibiotics to price regulated antibiotics (notified under DPCO 2013). We argue that policies on price control need to be complemented with continuous monitoring of market behavior to have a measurable and long-term impact.

**Supplementary Information:**

The online version contains supplementary material available at 10.1186/s40545-022-00466-4.

## Background

The World Health Assembly approved a resolution for transparency in medicine prices in 2019 urging governments to monitor the impact of pricing policies on the affordability and accessibility of medical products[1]. In LMICs, medicines account for 20–60% of total health care expenditure, with nearly 90% of the population purchasing medicines through out-of-pocket payments [2]. In countries with a dominant private market, the availability of essential drugs per se is a relatively smaller issue, but affordability continues to be a major challenge owing to the high prices of medicines targeting both acute and chronic conditions. A large segment of the population in LMICs was observed to be unable to afford monthly treatment costs for medicines meant for three common non-communicable diseases (NCD) conditions in the private sector [3]. Another study of cardiovascular disease (CVD) medicines in 18 countries suggested that medicines were unaffordable for some patients who purchased them from a private pharmacy [4]. India is a leading producer and exporter of generic medicines and has one of the lowest medicine prices in the world. Yet, due to a lack of financial risk protection mechanisms at the country level, the share of households’ in drug spending is as high as 90%, whereas the governments contribute only 10% [5].

National governments adopt various policy instruments to address market imperfections to control medicine prices and expenditures. Price regulation is often employed to bring down pharmaceutical prices. The approaches for regulating medicine prices include controlling mark-ups, reference pricing, price negotiations, cost-plus-based pricing, value-based pricing, pricing through tenders and pooled procurement [2]. The choice of one approach over the other is generally made by a country after carefully considering national priorities, health financing mechanisms, regulatory landscape and health system context within the country. Most countries adopt a mix of policy instruments to strike a balance between health policy and industrial policy goals. India has a long history of price regulation (since 1962), through the Drug Price Control Orders (DPCO).

The latest DPCO, 2013 [6] was notified on 15 May 2013 for the implementation of the National Pharmaceutical Pricing Policy (NPPP), 2012 [7]. The NPPP’s objective was ‘to put in place a regulatory framework for pricing of drugs to ensure availability of essential medicines at reasonable prices even while offering adequate opportunity for innovation and competition to support the pharmaceutical industry. As per the DPCO, 2013 all the drugs under the National List of Essential Medicines (*n* = 348) notified by the Ministry of Health and Family Welfare were brought under price control. DPCO 2013 set the price ceilings for these medicines averaging the existing market prices (of all brands that have a market share of 1% or greater) and adding a 16 per cent retailer’s margin to it. In addition, the brands priced below the ceiling price were required to maintain the prices at current levels, whereas the brands priced above the price cap had to reduce their prices.

Previous research from India on an antihyperlipidemic drug—atorvastatin—the price for which was regulated under DPCO 2013 suggested that bringing the drug under price control improved the relative sales of atorvastatin in the country [8] in comparison to non-price controlled statins. However, Meng et al. reported that implementation of retail price controls in isolation was not effective in controlling drug expenditures in Korea, as medicine utilization was the determining factor for achieving the same, more than the prices [9]. Similarly, Bhaskarabhatla et al. reported that price control of metformin—an antidiabetic medicine—notified under DPCO 2013 resulted in a modest improvement in its sales [10].

We argue that a majority of medicines are therapeutically substitutable at the formulation level within a therapeutic segment depending upon the clinical condition, and hence any policy measure that selectively targets some and not all medicines within a therapeutic segment may lead to unintended consequences. For example, Emma [11] reported an exit of local firms from the price-controlled molecule market on account of the DPCO 2013 in India, though they continued to produce non-price-controlled formulations of the same molecule.

We also recognize that much of the previous research on the impact of DPCO 2013 on medicine sales have been restricted to single formulations [10, 12] and over shorter time frames. The core objective of this research was to measure the long-term impact of DPCO 2013 on an entire therapeutic segment of systemic antibiotics, using a quasi-experimental research design and utilizing the latest available private sector pharmaceutical sales data. We chose the segment systemic antibiotics as India has a high burden of infectious diseases and a huge market for antibiotics.

## Methods

### Data

We utilised the Indian pharmaceutical sales data set PharmaTrac [13] which is collected by a market research company AIOCD-AWACS which is a joint venture between All Indian Origin Chemists and Distributors Ltd (AIOCD Ltd) and Trikaal Mediinfotech Pvt Ltd. from a panel of around 18,000 stockists spread across 23 different regions in India. The stockists are selected after carrying out a census to understand the total number of pharmaceutical companies in the state and then selecting those stockists that account for at least 25 per cent of their turnover. The pharmaceutical sales data are compiled and extracted every month from the computers of the selected stockists using the software. These data are then extrapolated to reflect the overall medicine sales in the private sector in India using companywise and statewise projection factors.

Pharmaceuticals in the data set are organised according to the anatomical therapeutic chemical (ATC) classification of the European Pharmaceutical Market Research Association (EphMRA). This classification was used to identify the private market for systemic antibiotics in the country. A total of 54 unique strengths and dosage forms of antibiotics were considered for the policy impact evaluation (see Additional file 1: Table S1) which were notified for price regulation under DPCO 2013. The notifications for the price ceiling were staggered between June 2013 (first) and December 2014 (final). The data do not capture medicines consumed in the government facilities, our analysis, therefore, focuses exclusively on the impact of the DPCO 2013 on private sector antibiotic utilisation.

### Intervention

The intervention under study is the Drug Price Control Order 2013 (DPCO, 2013) [6] which was notified on 15th May 2013 for the implementation of the National Pharmaceutical Pricing Policy (NPPP), 2012 [7] by the National Pharmaceutical Pricing Authority (NPPA), Ministry of Chemical and Fertilizers. The National Pharmaceutical Pricing Policy (NPPP), 2012 laid down three criteria for price control: (1) regulation of prices based on ‘essentiality of drugs’ (i.e., formulations as listed under the National List of Essential Medicines (NLEM) [14] notified by the Ministry of Health and Family Welfare, (2) control of formulation prices only and (3) market-based pricing.

The DPCO 2013 notifications for price control of antibiotics based on NLEM 2011 were staggered over a period of 19 months—the first notification was released in June 2013 and the final one in December 2014. However, the manufacturers were allowed a period of 45 days to comply with the notification and modify maximum retail prices (MRP) on the packs of medicine under notification for implementation reasons. We, therefore, considered the period from June 2013 to January 2015 as the implementation period.

It may be noted that the Government of India released another set of notifications in 2016 based on a new NLEM, 2015. In addition, the Government notified another policy to bring some of the antibiotics under a new clause ‘H1’ underlying the Indian Drugs and Cosmetics Rules, 1945 which required pharmacists to dispense these antibiotics only upon the production of a prescription from a Registered Medical Practitioner. However, the present analysis is confined only to the 2013 policy intervention. All antibiotic formulations that were influenced by the latter two interventions were excluded from the current analysis to avoid the confounding effects of multiple interventions. The analysis was, therefore, limited to 54 antibiotic formulations. All the antibiotic formulations included in the analysis along with their price ceiling notifications are provided in Additional file 1: Table S1. Another advantage of limiting the scope of the present analysis to the 2013 intervention was that we had sufficient data points available to us in both the pre- and post-intervention periods, independent of other interventions targeted at the medicines under study and their confounding impacts thereof.

### Outcome measures

The primary outcome measure is the percentage change (increase or decrease) in the sales volume of the antibiotics under DPCO 2013, measured in standard units (SUs). SU is defined as the smallest dose of formulation (one tablet or capsule for oral solids, one vial or ampoule for injectable). We computed sales volume in SUs of all dosage forms and strengths of antibiotics under the price regulation. We used the logarithmic form of the sales volumes to examine changes in sales volumes before and after the implementation of the DPCO 2013. Sales volume was considered as a proxy for antibiotic utilisation.

### Research design

We used interrupted time series, a quasi-experimental research design to capture the impact of price regulation on the utilization of antibiotics (notified under DPCO in June 2013) [3].

### Statistical analysis

We used interrupted time series (ITS) analysis, a quasi-experimental research design for the present study. ITS is commonly used to study the impact of policies by comparing pre-intervention trends with post-intervention trends, especially for health-related interventions [15–18].

We performed Interrupted Time Series Analysis (ITS) on the data spanning 132 month period that was distributed into two segments, pre-intervention and post-intervention period, based on notifications. The pre-intervention period referred to the period from January 2008 to May 2013 and the post-intervention period referred to the period from February 2014 to December 2018. The period of price ceiling notifications from June 2013 to January 2014 was considered the implementation period and, therefore, excluded from the analysis. Interrupted time series analysis was undertaken to detect the (a) pre-intervention level and trend, (b) post-intervention level and trend change in antibiotics utilisation.

The dependent variable (Y_t_) was the ‘logarithm of sales volume’ of all antibiotics under price control. The ‘Time’ factor appeared as an independent variable. We fitted a least square regression line to the two segments of the continuous variable time and introduced two variables to estimate the immediate level change after the intervention (variable name: intervention) and the trend change (variable name: time after intervention), respectively (see Eq. 1). The variable ‘intervention’ was ‘0’ for the pre-intervention period and ‘1’ for the post-intervention. Time after the intervention was incorporated as a continuous variable in the post-intervention period (model 1).

The interrupted time series analysis helped us to statistically determine the change in the intercept and the slope coefficients between the pre- and post-intervention period (α: measures the **base level** of the outcome at the beginning of the series; β1: estimates the **base trend;** β2: estimates the **change in level** in the post‐intervention segment; β3: estimates the **change in trend** in the post‐intervention segment).

A dummy (*d*) was introduced to factor in the seasonality of antibiotic use. The variable took the value 1 underpinning 3 months period—August–September–October each year. The choice of the seasonal dummy is consistent with the findings of earlier studies [19]:

1 $$Y_{{\text{t}}} = {\alpha} + {\beta}_{{1}} {\text{time}}_{{\text{t}}} + {\beta}_{{{2} }} {\text{intervention}}_{{\text{t}}} + {\beta}_{{{3} }} {\text{time after intervention}}_{{\text{t}}} + \, d \, + \epsilon_{t}$$

Furthermore, a counterfactual was introduced into the model to assess the outcome in absence of the intervention under study (DPCO 2013). It was assumed that in the absence of the price ceiling notification, the pre-intervention trend of antibiotic consumption would have remained unchanged in the post-intervention period (represented in the figure by a dotted line).

Since the antibiotic sales data were time-series in nature, we checked the regression model for autocorrelation using Durbin–Watson statistic, autocorrelation (ac) and partial autocorrelation (pac) estimates, and plots of the residuals (see Additional file 2). We detected first-order autocorrelation in our model and, therefore, altered it to the Prais–Winsten model (model 2) which makes use of the generalized least-squares method to estimate the parameters.

As part of sensitivity analysis, we ran another model using ‘market share’ of antibiotics under DPCO 2013 to the total antibiotic market as an outcome measure to understand the relative change in the market share of antibiotics under ‘DPCO 2013’ versus ‘not under DPCO 2013’ (model 3) to examine if there had been a switch in sales between price regulated and non-price regulated antibiotic formulations in response to the policy intervention. Prais–Winsten model was also used and reported in model 3, since first-order autocorrelation was detected. All analyses were carried out using Stata software version 14.

### Ethics

The study did not require primary data collection. It uses secondary data on pharmaceuticals and, therefore, did not require ethical clearances.

## Results

### Descriptive statistics

In absolute terms, India’s antibiotic consumption doubled between 2008 and 2018 (Table 1). In 2018, around 535 companies produced antibiotics worth INR 140 billion and the share of antibiotics as a proportion of overall medicine sales was nearly 15 percent.

**Table 1:** Descriptive statistics of the antibiotics market in India

Market	2008	2013	2018
Total Medicine Sales (INR Billions)	410	790	1290
Antibiotics Sales (INR Billions)	74	113	140
Price Regulated Antibiotics Sales (Value INR Billions; % in parentheses)	26.2 (35%)	36.3 (32%)	40.6 (29%)
Price Regulated Antibiotics Sales (Volume in million SUs; % in parentheses)	4495 (45%)	5873 (46%)	5855 (49%)
Number of Companies Selling Antibiotics	535	401	535

Millions, billions and percentages rounded off

*INR* Indian National Rupee

### Interrupted time series analysis results

The results from the interrupted time series analysis (Model 1, Table 2), suggest that in the pre-intervention period, the average monthly sale of antibiotics under DPCO 2013 increased by 0.4 per cent (*p* < 0.01), whereas post-intervention there was an immediate increase (level change) of 0.8 per cent (*p* > 0.05) and a sustained decline (trend change) of 0.3 per cent (*p* < 0.01) in comparison to the pre-intervention level and trend. However, we detected first-order auto-correlation in Model 1 (Additional file 2: Figs. S1, S2). We used Prais–Winsten model (Model 2, Table 2) to correct autocorrelation. The revised estimates suggest that post-intervention there was an immediate reduction (level change) in the sales of antibiotics under DPCO 2013 by 3.7% (*P* > 0.05), followed by a sustained decline (trend change) of 0.3% (*P* > 0.05) as compared to the pre-intervention scenario, but both changes were statistically insignificant.

**Table 2:** Interrupted time series analysis results for utilization of antibiotics under price control

Variable	MODEL 1		MODEL 2		MODEL 3	
	Coefficient	*p* value (95%CI)	Coefficient	*p* value (95%CI)	Coefficient	*p* value (95%CI)
Time	0.004	0.000 (0.003–0.005)	0.005	0.000 (0.002–0.007)	0.81	0.045 (0.001–0.161)
Intervention (level change)	− 0.008	0.809 (− 0.059–0.076)	− 0.037	0.517 (− 0.152–0.077)	− 5.795	0.000 (− 8.243– − 3.347)
Time after intervention (trend change)	− 0.003	0.002 (− 0.005–− 0.001)	− 0.003	0.100 (− 0.007–0.000)	0.095	0.257 (0.070–0.262)
Seasonal dummy	0.208	0.000 (0.169–0.247)	0.125	0.000 (0.083–0.167)		
Const.	19.63	0.000 (19.593–19.683)	19.63	0.000 (19.46–19.71)	43.13	0.000 (39.806–46.460)
Number of observations	112 (pre-intervention: 65 and post-intervention: 47)		112 (pre-intervention: 65 and post-intervention: 47)		112 (pre-intervention: 65 and post-intervention: 47)	
*R*^2^	0.7208		0.987		0.625	

**Fig. 1:**
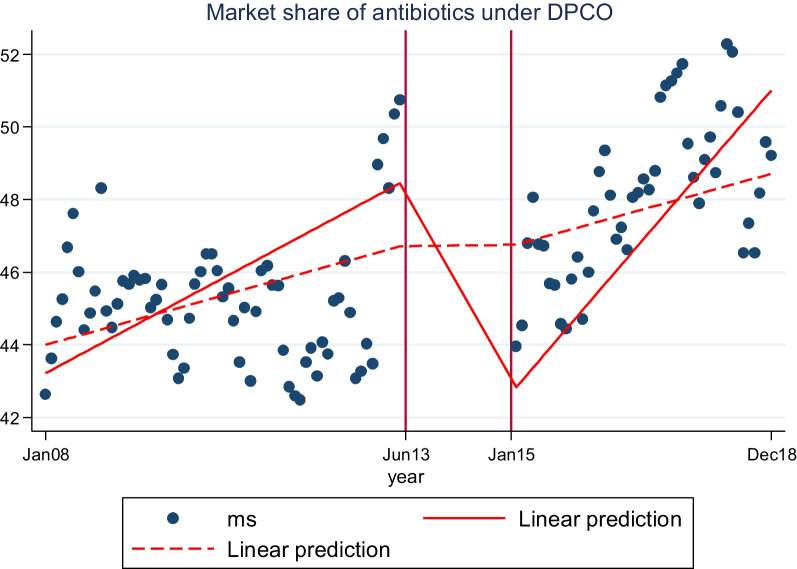
Fitted values of market share of price-regulated antibiotics—actual and counterfactual

Furthermore, as part of the sensitivity analysis, we used the ‘market share’ of antibiotics under DPCO 2013 in the overall antibiotics market as the outcome variable to examine whether there was a switch in antibiotic sales between non-price controlled antibiotics and those under DPCO 2013 (Model 3, Table 2, Fig. 1). Results from model 3 suggest that post-intervention the average monthly market share of the antibiotic under DPCO 2013 fell by more than 579% (*P* < 0.05) (level change) followed by a sustained increase of 9.5% (*P* > 0.05) (trend change) as compared with the pre-intervention trend.

Table 3 highlights results from interrupted time series analysis conducted on subtherapeutic categories of the antibiotics under DPCO 2013. The estimates suggest that post-intervention, antibiotics belonging to the therapeutic class broad-spectrum penicillins, cephalosporins, macrolides, trimethoprim and other antibacterials witnessed a sustained reduction in sales while aminoglycoside, narrow spectrum penicillin and fluoroquinolones witnessed increased sales in comparison to the pre-intervention trend. However, the trend change was observed to be statistically significant only for the segments—macrolides, trimethoprim and other antibacterials.

**Table 3:** Interrupted time series analysis results for utilization of antibiotics under price control, by sub-therapeutic categories

Subtherapeutic categories	Time (*p* value)	Intervention (*p* value)	Time after intervention (*p* value)	Constant (*p* value)	Goodness of fit (*R*^2^)
Aminoglycosides	− 0.001 (0.41)	− 0.170 (0.08)	0.002 (0.42)	14.45 (0.00)	0.94
Broad spectrum penicillin	0.009 (0.00)	0.238 (0.00)	− 0.002 (0.35)	17.43 (0.00)	0.98
Narrow spectrum penicillin	0.002 (0.93)	0.355 (0.44)	0.021 (0.68)	12.08 (0.00)	0.40
Cephalosporins	0.006 (0.00)	− 0.011 (0.88)	− 0.006 (0.56)	17.98 (0.00)	0.98
Fluroquinolones	− 0.001 (0.39)	− 0.065 (0.43)	0.002 (0.50)	18.19 (0.00)	0.97
Macrolides	0.005 (0.00)	0.180 (0.02)	− 0.007 (0.01)	17.30 (0.00)	0.98
Tetracyclines	− 0.014 (0.00)	− 2.11 (0.00)	− 0.004 (0.28)	18.83 (0.00)	0.97
Trimethoprim and combinations	0.002 (0.73)	0.291 (0.10)	− 0.024 (0.00)	17.66 (0.00)	0.90
Other antibacterials	0.006 (0.00)	0.878 (0.00)	− 0.010 (0.00)	10.56 (0.00)	0.88

## Discussion

To the best of our knowledge, this is the first report on the impact of DPCO 2013 on antibiotic utilization in India. Our analyses reveal that the effect of price control on antibiotic utilization was modest and limited. Post-intervention (after implementation of DPCO 2013) there was a reduction in the sales of antibiotics under DPCO 2013 by 3.7% which was followed by a sustained decline of 0.3%, as compared to the pre-intervention level and trend. However, both changes were statistically insignificant.

The limited impact on overall antibiotic sales could be explained through the differential impact of DCPO 2013 on different therapeutic categories of antibiotics available in the market. For example, we observed that antibiotics belonging to the therapeutic classes broad-spectrum penicillin, cephalosporins, macrolides, trimethoprim and other antibacterials witnessed a sustained reduction in sales in the post-intervention period, whereas narrow spectrum penicillin, fluoroquinolones and aminoglycosides saw increased sales, though statistically non-significant. Another possible explanation could be the design of the policy and the implementation challenges associated with it. The DPCO 2013 was based on the Essential Medicine List 2011 notified by the Ministry of Health and Family Welfare which has a limited number of antibiotic formulations, which are not necessarily the most prescribed antibiotics in the country’s private sector. In addition, the demand for antibiotics in the market is not only driven by the changing epidemiological pattern but also influenced by market imperfections. For instance, manufacturers can influence prescriber behavior through medical representatives and can shift antibiotic prescriptions toward molecules that are not under price regulation [20, 21].

Earlier, using a different methodological approach Sahay et al. reported that DPCO 2013 had a variable impact on the sales volume of medicines under price control. They reported that for a majority of the molecules (52) price regulation had a negative impact on their sales volume, while a few molecules (37) witnessed an increase in sales especially those prescribed for chronic illnesses [22]. Another independent evaluation reported that DPCO 2013 had resulted in reduced sales of medicines manufactured by small local generics manufacturers. Such medicines were observed to have had around a 14.5 per cent reduction in the market share and around a 5.3 per cent decline in sales [11].

However, these studies did not examine the dynamic changes in medicine utilisation resulting from the price regulation on a therapeutic segment where medicines are highly substitutable such as antibiotics. We postulate that since a majority of antibiotics are substitutable within a therapeutic class, price controls or price reductions should potentially lead prescribers and consumers to switch toward price-controlled antibiotics (cheaper formulations) from non-price-controlled ones (expensive formulations). Our sensitivity analysis using the ‘market share’ of antibiotics under DPCO 2013 to the overall antibiotics market as the outcome variable (model 3, Table 2) demonstrates the switch in sales toward price-regulated antibiotics from non-price-regulated antibiotics. Our estimates from model 3 suggest that the average monthly market share of antibiotics under DPCO 2013 increased by 9.5% in comparison to the pre-intervention period over the duration of the study. This empirical evidence also reflects the clinical practice and market behavior, highlighting that patients and prescribers switch toward cheaper alternatives. Our previous research on statins also found that price regulation led to a relative increase in the sales of the regulated Atorvastatin in the statins market in India [12].

However, price regulation alone does not guarantee a reduction in the overall expenditure on medicines or treatment costs. Previous research suggests that there could be unintended effects of price control policies. For example, Yoo et al. reported that the implementation of the drug price control policy, did reduce the expenditure by US$ − 1.51, (− 10.2 per cent) in Korea, but it also led to an increased average number of drugs prescribed per month, leading to overutilization and use of prohibited combinations [18].

Our study has some limitations. We did not assess the effect of price regulation on medicines other than antibiotics hence our findings are not representative of other therapeutic segments under price control. In addition, our study did not quantify the extent of price reduction on antibiotics. Finally, the scope of the research was limited to the private sector market and we did not evaluate the impact of DPCO 2013 on the public sector antibiotic utilization because of a lack of nationally representative public sector data on antibiotics. This is, however, not a particularly significant issue as 85–90 percent of prescriptions in the country occur in the private sector [23].

## Conclusions

Our analysis suggests that DPCO 2013 had a limited impact in increasing utilization of all antibiotics under price regulation but there was a switch from non-price controlled antibiotics to price regulated antibiotics. It may be argued that in India and other market-oriented low and middle-income countries, where a significant proportion of the population seeks care in the private sector, price regulation is critical to contain pharmaceutical expenditure to ensure affordable healthcare. However, price regulation is not without unintended effects, hence continuous monitoring of sales and marketing practices of the manufacturers, and prescribers’ behavior are equally important. Further research is needed to investigate the impact of DPCO 2013 on other therapeutic markets.

## Supplementary Information

**Additional file 1: Table S1. **Antibiotics in NLEM, 2011 and their price-ceiling notification dates by NPPA.

**Additional file 2. **Autocorrelation (AC) and Partial Autocorrelation (PAC) plots of residuals for model 1.

## Data Availability

The data that support the findings of this study are available from AIOCD AWACS a pharmaceutical market research organization but restrictions apply to the availability of these data, which were used under license for the current study, and so are not publicly available. Data are, however, available from the authors upon reasonable request and with permission of AIOCD AWACS https://aiocdawacs.com/(S(1it2biqinendln3ovlglqu3m))/ProductDetail.aspx.
