# Supplementary material for: Evaluating the impact of price regulation (Drug Price Control Order 2013) on antibiotic sales in India: a quasi-experimental analysis, 2008–2018
Source: J Pharm Policy Pract. 2022 Oct 22;15:68. doi: 10.1186/s40545-022-00466-4 (PMC9587621; doi:10.1186/s40545-022-00466-4)
Supplement: Supplementary file 1 — Additional file 1: Table S1. Antibiotics in NLEM, 2011 and their price-ceiling notification dates by NPPA. [file 40545_2022_466_MOESM1_ESM.docx]

**Additional file 1**

**Table S1. Antibiotics in NLEM, 2011 and their price-ceiling notification dates by NPPA**

| **Section** | **Section-code** | **Sub Section** | **Sub Section-code** | **Sub Section2** | **Sub Section2-code** | **Medicine Name** | **coding done in stata/ Remarks** | **Strength** | **Mode of Administration** | **Date of Notification** |
| --- | --- | --- | --- | --- | --- | --- | --- | --- | --- | --- |
| Anti-infective Medicines | 6 | Antibacterials | 6.2 | Other antibacterials | 6.2.2 | Amikacin |  | 250 mg/ 2 ml | Injection | 21-06-2013 |
| Anti-infective Medicines | 6 | Antibacterials | 6.2 | Beta lactam medicines | 6.2.1 | Amoxicillin |  | 125 mg/ 5 ml | Powder for suspension | 21-06-2013 |
| Anti-infective Medicines | 6 | Antibacterials | 6.2 | Beta lactam medicines | 6.2.1 | Amoxicillin |  | 250 mg | Capsules | 05-07-2013 |
| Anti-infective Medicines | 6 | Antibacterials | 6.2 | Beta lactam medicines | 6.2.1 | Amoxicillin |  | 500 mg | Capsules | 21-06-2013 |
| Anti-infective Medicines | 6 | Antibacterials | 6.2 | Beta lactam medicines | 6.2.1 | Amoxicillin + Clavulinic acid |  | 625 mg | Tablets | 28-06-2013 |
| Anti-infective Medicines | 6 | Antibacterials | 6.2 | Beta lactam medicines | 6.2.1 | Amoxicillin + Clavulinic acid |  | 228.5 mg/ 5ml | Powder for suspension | 28-06-2013 |
| Anti-infective Medicines | 6 | Antibacterials | 6.2 | Beta lactam medicines | 6.2.1 | Amoxicillin + Clavulinic acid |  | 600 mg | Injection | 28-06-2013 |
| Anti-infective Medicines | 6 | Antibacterials | 6.2 | Beta lactam medicines | 6.2.1 | Amoxicillin + Clavulinic acid |  | 1.2 gm | Injection | 28-06-2013 |
| Anti-infective Medicines | 6 | Antibacterials | 6.2 | Beta lactam medicines | 6.2.1 | Ampicillin |  | 250 mg | Capsules | 05-07-2013 |
| Anti-infective Medicines | 6 | Antibacterials | 6.2 | Beta lactam medicines | 6.2.1 | Ampicillin |  | 500 mg | Capsules | 21-06-2013 |
| Anti-infective Medicines | 6 | Antibacterials | 6.2 | Beta lactam medicines | 6.2.1 | Ampicillin |  | 125 mg/ 5 ml | Powder for suspension | 14-06-2013 |
| Anti-infective Medicines | 6 | Antibacterials | 6.2 | Beta lactam medicines | 6.2.1 | Ampicillin |  | 500 mg | Injection | 21-06-2013 |
| Anti-infective Medicines | 6 | Antibacterials | 6.2 | Other antibacterials | 6.2.2 | Azithromycin |  | 100 mg | Tablets | 05-07-2013 |
| Anti-infective Medicines | 6 | Antibacterials | 6.2 | Other antibacterials | 6.2.2 | Azithromycin |  | 250 mg | Tablets | 05-07-2013 |
| Anti-infective Medicines | 6 | Antibacterials | 6.2 | Other antibacterials | 6.2.2 | Azithromycin |  | 500 mg | Tablets | 14-06-2013 |
| Anti-infective Medicines | 6 | Antibacterials | 6.2 | Other antibacterials | 6.2.2 | Azithromycin |  | 100 mg/ 5 ml | Suspension | 22-07-2013 |
| Anti-infective Medicines | 6 | Antibacterials | 6.2 | Other antibacterials | 6.2.2 | Azithromycin |  | 500 mg | Injection | 14-06-2013 |
| Anti-infective Medicines | 6 | Antibacterials | 6.2 | Beta lactam medicines | 6.2.1 | Benzathine Benzylpenicilin |  | 6 Lacs Units | Injection | 21-06-2013 |
| Anti-infective Medicines | 6 | Antibacterials | 6.2 | Beta lactam medicines | 6.2.1 | Benzathine Benzylpenicilin |  | 12 Lacs Units | Injection | 27-03-2014 |
| Anti-infective Medicines | 6 | Antibacterials | 6.2 | Beta lactam medicines | 6.2.1 | Cefixime |  | 100 mg | Tablets | 21-06-2013 |
| Anti-infective Medicines | 6 | Antibacterials | 6.2 | Beta lactam medicines | 6.2.1 | Cefixime |  | 200 mg | Tablets | 22-07-2013 |
| Anti-infective Medicines | 6 | Antibacterials | 6.2 | Beta lactam medicines | 6.2.1 | Cefotaxime |  | 125 mg | Injection | 20-08-2014 |
| Anti-infective Medicines | 6 | Antibacterials | 6.2 | Beta lactam medicines | 6.2.1 | Cefotaxime |  | 250 mg | Injection | 15-09-2014 |
| Anti-infective Medicines | 6 | Antibacterials | 6.2 | Beta lactam medicines | 6.2.1 | Cefotaxime |  | 500 mg | Injection | 15-09-2014 |
| Anti-infective Medicines | 6 | Antibacterials | 6.2 | Beta lactam medicines | 6.2.1 | Ceftazidine |  | 250 mg | Injection | 14-06-2013 |
| Anti-infective Medicines | 6 | Antibacterials | 6.2 | Beta lactam medicines | 6.2.1 | Ceftazidine |  | 1 gm | Injection | 21-06-2013 |
| Anti-infective Medicines | 6 | Antibacterials | 6.2 | Beta lactam medicines | 6.2.1 | Ceftriaxone |  | 250 mg | Injection | 21-06-2013 |
| Anti-infective Medicines | 6 | Antibacterials | 6.2 | Beta lactam medicines | 6.2.1 | Ceftriaxone |  | 1 gm | Injection | 22-07-2013 |
| Anti-infective Medicines | 6 | Antibacterials | 6.2 | Beta lactam medicines | 6.2.1 | Cephalexin |  | 125 mg/ 5 ml | Syrup | 07-05-2013 |
| Anti-infective Medicines | 6 | Antibacterials | 6.2 | Beta lactam medicines | 6.2.1 | Cephalexin |  | 250 mg | Capsules | 05-07-2013 |
| Anti-infective Medicines | 6 | Antibacterials | 6.2 | Beta lactam medicines | 6.2.1 | Cephalexin |  | 500 mg | Capsules | 05-07-2013 |
| Anti-infective Medicines | 6 | Antibacterials | 6.2 | Other antibacterials | 6.2.2 | Ciprofloxacin Hydrochloride |  | 200 mg / 100 ml | Injection | 28-04-2014 |
| Anti-infective Medicines | 6 | Antibacterials | 6.2 | Other antibacterials | 6.2.2 | Ciprofloxacin Hydrochloride |  | 250 mg | Tablets | 15-09-2014 |
| Anti-infective Medicines | 6 | Antibacterials | 6.2 | Other antibacterials | 6.2.2 | Ciprofloxacin Hydrochloride |  | 500 mg | Tablets | 15-09-2014 |
| Anti-infective Medicines | 6 | Antibacterials | 6.2 | Beta lactam medicines | 6.2.1 | Cloxacillin |  | 250 mg | Capsules | 10-12-2014 |
| Anti-infective Medicines | 6 | Antibacterials | 6.2 | Beta lactam medicines | 6.2.1 | Cloxacillin |  | 500 mg | Capsules | 15-09-2014 |
| Anti-infective Medicines | 6 | Antibacterials | 6.2 | Beta lactam medicines | 6.2.1 | Cloxacillin |  | 250 mg | Injection | 15-09-2014 |
| Anti-infective Medicines | 6 | Antibacterials | 6.2 | Other antibacterials | 6.2.2 | Co-Trimoxazole ( Trimethoprim + Sulphamethoxazole |  | 80+400 mg | Tablets | 15-09-2014 |
| Anti-infective Medicines | 6 | Antibacterials | 6.2 | Other antibacterials | 6.2.2 | Co-Trimoxazole ( Trimethoprim + Sulphamethoxazole |  | 160+800 mg | Tablets | 28-04-2014 |
| Anti-infective Medicines | 6 | Antibacterials | 6.2 | Other antibacterials | 6.2.2 | Co-Trimoxazole ( Trimethoprim + Sulphamethoxazole |  | 40+200 mg/ 5 ml | Suspension | 28-04-2014 |
| Anti-infective Medicines | 6 | Antibacterials | 6.2 | Other antibacterials | 6.2.2 | Doxycyclin |  | 100 mg | Tablets | 28-04-2014 |
| Anti-infective Medicines | 6 | Antibacterials | 6.2 | Other antibacterials | 6.2.2 | Erythromycin Estolate |  | 125 mg/ 5 ml | Syrup | 10-06-2013 |
| Anti-infective Medicines | 6 | Antibacterials | 6.2 | Other antibacterials | 6.2.2 | Erythromycin Estolate |  | 250 mg | Tablets | 10-06-2013 |
| Anti-infective Medicines | 6 | Antibacterials | 6.2 | Other antibacterials | 6.2.2 | Erythromycin Estolate |  | 500 mg | Tablets | 10-06-2013 |
| Anti-infective Medicines | 6 | Antibacterials | 6.2 | Other antibacterials | 6.2.2 | Gentamicin |  | 10 mg/ ml | Injection | 10-12-2014 |
| Anti-infective Medicines | 6 | Antibacterials | 6.2 | Other antibacterials | 6.2.2 | Gentamicin |  | 40 mg/ ml | Injection | 28-04-2014 |
| Anti-infective Medicines | 6 | Antibacterials | 6.2 | Other antibacterials | 6.2.2 | Metronidazole |  | 200 mg | Tablets | 10-06-2013 |
| Anti-infective Medicines | 6 | Antibacterials | 6.2 | Other antibacterials | 6.2.2 | Metronidazole |  | 400 mg | Tablets | 10-06-2013 |
| Anti-infective Medicines | 6 | Antibacterials | 6.2 | Other antibacterials | 6.2.2 | Metronidazole |  | 500 mg/ 100 ml | Injection | 10-06-2013 |
| Anti-infective Medicines | 6 | Antibacterials | 6.2 | Other antibacterials | 6.2.2 | Metronidazole |  | 100 mg/ 5 ml | Syrup | 02-04-2014 |
| Anti-infective Medicines | 6 | Antibacterials | 6.2 | Other antibacterials | 6.2.2 | Nitrofurantoin |  | 100 mg | Tablets | 28-06-2013 |
| Anti-infective Medicines | 6 | Antibacterials | 6.2 | Other antibacterials | 6.2.2 | Sulphadiazine |  | 500 mg | Tablets | 15-09-2014 |
| Anti-infective Medicines | 6 | Antibacterials | 6.2 | Other antibacterials | 6.2.2 | Vancomycin Hydrochloride |  | 500 mg | Injection | 14-06-2013 |
| Anti-infective Medicines | 6 | Antibacterials | 6.2 | Other antibacterials | 6.2.2 | Vancomycin Hydrochloride |  | 1 gm | Injection | 14-06-2013 |
