# Supplementary material for: Evaluating the impact of price regulation (Drug Price Control Order 2013) on antibiotic sales in India: a quasi-experimental analysis, 2008–2018
Source: J Pharm Policy Pract. 2022 Oct 22;15:68. doi: 10.1186/s40545-022-00466-4 (PMC9587621; doi:10.1186/s40545-022-00466-4)
Supplement: Supplementary file 2 — Additional file 2. Autocorrelation (AC) and Partial Autocorrelation (PAC) plots of residuals for model 1. [file 40545_2022_466_MOESM2_ESM.docx]

Additional file 2

Autocorrelation (AC) and Partial Autocorrelation (PAC) plots of residuals for model 1

Figure S1. AC plot

Figure S2. PAC plot
